# Fast Dissolving Sublingual Films of Ondansetron Hydrochloride: Effect of Additives on *in vitro* Drug Release and Mucosal Permeation

**DOI:** 10.4103/0975-1483.66790

**Published:** 2010

**Authors:** M Koland, VP Sandeep, NR Charyulu

**Affiliations:** *Department of Pharmaceutics, Nitte Gulabi Shetty Memorial Institute of Pharmaceutical Sciences, Mangalore - 574 160, Karnataka, India*

**Keywords:** Fast dissolving films, ondansetron, polyvinyl alcohol, solvent casting, sublingual films

## Abstract

Ondansetron hydrochloride, a 5 HT3 antagonist is a powerful antiemetic drug which has oral bioavailability of 60% due to hepatic first pass metabolism and has a short half-life of 5 h. To overcome the above draw back, the present study was carried out to formulate and evaluate fast dissolving films of ondansetron hydrochloride for sublingual administration. The films were prepared from polymers such as polyvinylalcohol, polyvinyl pyrrolidone, Carbopol 934P in different ratios by solvent casting method. Propylene glycol or PEG 400 as plasticizers and mannitol or sodium saccharin as sweeteners were also included. The IR spectral studies showed no interaction between drug and polymer or with other additives. Satisfactory results were obtained when subjected to physico-chemical tests such as uniformity of weight, thickness, surface pH, folding endurance, uniformity of drug content, swelling index, bioadhesive strength, and tensile strength. Films were also subjected to *in vitro* drug release studies by using USP dissolution apparatus. *Ex vivo* drug permeation studies were carried out using porcine membrane model. *In vitro* release studies indicated 81–96% release within 7 min and 66–80% within 7 min during *ex vivo* studies. Drug permeation of 66–77% was observed through porcine mucosa within 40 min. Higher percentage of drug release was observed from films containing the sweeteners. The stability studies conducted for a period of 8 weeks showed no appreciable change in drug content, surface pH, and *in vitro* drug release.

## INTRODUCTION

Fast mouth dissolving films have become popular as a new delivery system because they are easy to administer and sudden-onset of drug action is possible as the films are taken through the sublingual route. Since the sublingual mucosa is relatively permeable because of thin membrane and is highly perfused, rapid drug absorption and instant bioavailability is possible and this leads to quick-onset of drug action. Since the drug is directly absorbed into the systemic circulation, degradation in the gastrointestinal (GI) tract and first pass effect can be avoided.[1] Moreover, better patient compliance is expected, because this system does not require being swallowed as in the case of conventional tablet, and therefore beneficial in patients with dysphagia or difficulty in swallowing. The use of mucoadhesive polymers in the films will enable them to adhere to the sublingual mucosa for better retention and drug absorption.[2] Ondansetron, a 5HT_3_ antagonist is a potent antiemetic drug, which is used in control of nausea, vomiting associated with cancer chemotherapy. It exhibits only 60–70% of oral bioavailability because of first pass metabolism and has a relative short half-life of 3–5 h.[3] Studies have shown that ondansetron hydrochloride is well absorbed through the buccal or sublingual mucosa.[4] In view of all the above reasons, this study will be an attempt to optimize the therapeutic effect of ondansetron by formulating as fast mouth dissolving films for sublingual use.

## MATERIALS AND METHODS

### Materials

Ondansetron hydrochloride was obtained as a gift sample from Sun Pharma Pvt, Ltd., Ahemadabad. Carbopol 934P and Polyvinyl alcohol was obtained from CDH Laboratory. Polyvinyl pyrolidone K-30 was procured from Ozone International, Mumbai. Mannitol and saccharin sodium were laboratory grade ingredients from Loba Chemie, Mumbai.

### Preparation of fast dissolving ondansetron hydrochloride film

Fast dissolving films were prepared by solvent casting method[5] (Weinberger, 1987). Aqueous solution I was prepared by dissolving polymer in 20 mL hot water (80°C) with stirring to produce a clear solution and kept for 1 h to remove all the air bubbles.[6] In the case of the FC formulations, Carbopol 934P was first dissolved in a small portion of distilled water, neutralized with triethanolamine and then added to the cooled Poly Vinyl Alcohol (PVA) solution. Aqueous solution II was prepared by dissolving pure drug, sweetener, and plasticizer in specific proportion in distilled water. The aqueous solutions I and II were mixed and stirred for 1 h. The solutions were cast on to 9-cm diameter Petri dish and were dried in the oven at 45°C for 24 h. The films was carefully removed from the Petridish and checked for any imperfection and cut according to size required for testing (square film 2 cm length, 2 cm width) so that each film contained 4 mg of the drug. The samples were stored in a glass container maintained at temperature 30 °C and relative humidity 60% ± 5% until further analysis[Table 1].[2]

**Table 1:** Composition of ondansetron fast dissolving films

Ingredient (% w/w)	Formulation								
	FA			FB			FC		
	FA1	FA2	FA3	FB1	FB2	FB3	FC1	FC2	FC3
Ondansetron HCl	0.09	0.07	0.09	0.09	0.07	0.09	0.12	0.09	0.12
PVA	52.62	43.95	52.55	59.20	49.44	59.12	80.91	63.72	80.76
PVP	13.15	10.99	13.14	6.58	5.49	6.57	–	–	–
Carbopol	–	–	–	–	–	–	8.99	7.08	8.97
Propylene glycol	34.14	28.51	34.09	34.14	28.51	34.09	–	–	--
PEG-400	–	–	–	–	–	–	9.98	7.86	9.96
Mannitol	–	16.48	–	–	16.48	–	–	21.24	--
Sodium saccharin	–	–	0.13	–	–	0.13	–	–	0.18

### Determination of physicochemical parameters

The average weight each of 10 samples of each formulation was determined. The thickness of each of sample was measured using micrometer screw gauge at five locations, and the mean thicknesses were calculated.[6] The folding endurance was determined by repeatedly folding one film at the same place till it broke or folded up to 300 times which is considered satisfactory to reveal good film properties. The number of times the film could be folded at the same place without breaking gives the value of the folding endurance. The surface pH of films was determined to investigate the possible side effect because of change in pH *in vivo*, since an acidic or alkaline pH may cause irritation to buccal mucosa. The film to be tested was placed in a Petri dish and was moistened with 0.5 mL of distilled water and kept for 30 s. The pH was noted after bringing the electrode of the pH meter in contact with the surface of the formulation and allowing equilibrating for 1 min. The average of 10 determinations for each of the formulation was taken.[7] The results for all the films are shown in Table 2. The film formulations were also subjected to IR spectral studies to determine compatibility between drug and other components in the films.

**Table 2:** Physical characterization of film formulations

Formulation code		Weight* (mg)	Thickness* (mm)	Surface pH*	% Drug content*	Folding endurance*	Mucoadhesion time (sec)*
FA	FA_1_	51 ± 1.00	0.38 ± 0.015	6.63 ± 0.021	96.59 ± 2.32	>300	150
	FA_2_	60 ± 1.527	0.55 ± 0.081	6.54 ± 0.007	92.60 ± 4.23	>300	90
	FA_3_	55 ± 1.527	0.44 ± 0.052	6.45 ± 0.028	95.80 ± 3.27	>300	120
FB	FB_1_	65 ± 2.516	0.45 ± 0.06	6.49 ± 0.007	91.20 ± 4.13	>300	153
	FB_2_	75 ± 1.527	0.58 ± 0.008	6.20 ± 0.014	86.63 ± 7.04	>300	128
	FB_3_	67 ± 1.00	0.44 ± 0.009	6.32 ± 0.007	90.36 ± 8.95	>300	145
FC	FC_1_	80 ± 1.527	0.58 ± 0.010	7.08 ± 0.001	86.41 ± 5.34	>300	270
	FC_2_	95 ± 2.516	0.71 ± 0.015	6.81 ± 0.021	85.96 ± 5.77	>300	235
	FC_3_	83 ± 1.527	0.64 ± 0.032	7.04 ± 0.021	88.66 ± 2.56	>300	240

* Values represent the mean ± S.D and *n*=10 for weight and thickness and *n*=3 for others

### Measurement of swelling index

The studies for swelling index of the film were conducted in simulated salivary fluid of pH 6.75. The film sample (surface area 4 cm^2^) was weighed and placed in a preweighed stainless steel wire sieve of approximately 800-µm mesh. The mesh containing the film sample was submerged into 50 mL of simulated salivary medium contained in a mortar. At definite time interval (30 s), the stainless steel mesh was removed, excess moisture removed by carefully wiping with absorbent tissue and reweighed. Increase in weight of the film was determined at each time interval until a constant weight was observed.[8] The degree of swelling was calculated using the formula

$$\mathrm{SI}\;=\;\frac{W_{t}-W_{o}}{W_{o}},$$

where SI is the swelling index, *W*_t_ is the weight of the film at time ‘*t*’, and *W*_o_ is the weight of film at *t* = 0.

### Tensile strength measurement

This mechanical property was evaluated using Instron Universal Testing Instrument (model F. 4026), Instron Ltd., Japan, NITK, Surathkal) with a 5-kg load cell. Film strips in special dimension and free from air bubbles or physical imperfections were held between two clamps positioned at a distance of 3 cm. During measurement, the strips were pulled by the top clamp at a rate of 100 mm/min; the force and elongation were measured when the film broke. Results from film samples, which broke at and not between the clamps, were not included in the calculations. Measurements were run in triplicate for each film.

Two mechanical properties namely, tensile strength (TS) and percentage elongation were computed for the evaluation of the film. TS is the maximum stress applied to a point at which the film specimen breaks and can be computed from the applied load at rupture as a mean of three measurements and cross-sectional area of fractured film from the following equation.[6]

$$\mathrm{Tensile}\;\mathrm{strength}\;=\;\frac{\mathrm{Force}\;\mathrm{at}\;\mathrm{break}}{\mathrm{Initial}\;\mathrm{cross}-\mathrm{sectional}\;\mathrm{area}\;\mathrm{of}\;\mathrm{the}\;\mathrm{sample}\;({\mathrm{mm}}^{2})}$$

Percentage elongation can be obtained by following equation:

$$\%\mathrm{Elongation}\;\mathrm{at}\;\mathrm{break}\;=\;\frac{\mathrm{Increase}\;\mathrm{in}\;\mathrm{length}}{\mathrm{Original}\;\mathrm{length}}\;\times \;100$$

### Uniformity of drug content

This parameter was determined by dissolving one film of dimension 2 cm × 2 cm containing 4 mg of ondansetron hydrochloride by homogenization in 100 mL of stimulated saliva of pH 6.8 for 30 min with continuous shaking. From this, 10 mL was diluted to 50 mL with simulated salivary fluid. The absorbance was measured at 248 nm using an UV spectrometer. The experiments were carried out in triplicate for the films of all formulations and average values were recorded and given in Table 2.

### *In vitro* dissolution studies

Dissolution profile of fast dissolving films of ondansetron hydrochloride was carried out using USP type II (paddle apparatus) with 300 mL of simulated salivary fluid (pH 6.8) as dissolution medium maintained at 37 ± 0.5 °C. Medium was stirred at 100 rpm. Samples were withdrawn at every 30 s interval, replacing the same amount with the fresh medium.[2] Absorbance was determined by UV spectrophotometer at 248 nm.

### *Ex vivo* permeation studies through porcine oral mucosa

Permeation studies were carried using the modified Franz diffusion cell of internal diameter of 2.5 cm. Porcine oral mucosa was used as the model membrane. The buccal pouch of the freshly killed pig was procured from the local slaughter house. The buccal mucosa was excised and trimmed evenly from the sides and then washed in isotonic phosphate buffer of pH 6.6 and used immediately. The membrane was stabilized before mounting to remove the soluble components. The mucosa was mounted between the donor and receptor compartments. The receptor compartment was filled with 200 mL of isotonic phosphate buffer of pH 7.4 which was maintained at 37 °C ± 0.2 °C and the hydrodynamics were maintained by stirring with a magnetic bead at 50 rpm. One film of dimensions 2 cm × 2 cm and previously weighed was placed in intimate contact with the mucosal surface of the membrane that was previously moistened with a few drops of simulated saliva. The donor compartment was filled with 1 mL of simulated saliva of pH 6.8. Samples were withdrawn at suitable interval, replacing the same amount with the fresh medium.[9,10] The percentage of drug permeated was determined by measuring the absorbance in a UV–Visible spectrophotometer at 248 nm.

### *Ex vivo* mucoadhesion time and drug release

The *ex vivo* mucoadhesive time was performed by application of the film on freshly cut porcine buccal mucosa. The porcine tissues were fixed on the internal side of a beaker with cyano acrylate glue. The film was wetted with 50 µL of simulated saliva fluid and was pasted to the porcine buccal tissue by applying a light force with a fingertip for 20 s. The beaker was filled with 200 mL simulated saliva fluid and kept at 37 °C. After 2 min, a 50-rpm stirring rate was applied to simulate the buccal cavity environment and during the test, the time taken for the film to completely erode or detach from the mucosa was observed as the *ex vivo* mucoadhesion time. A 5-mL sample was withdrawn at every 30-s time interval, replacing the same amount with fresh medium.[11] After filtration, the amount of drug in the withdrawn samples was determined by UV spectrophotometer at 248 nm.

### Stability studies

The film formulations were also subjected to stability studies by storing them for 8 weeks under environmental conditions such as room temperature of 27 ± 2 °C/65% RH, oven temperature of 40 ± 2 °C/75% RH and in the refrigerator at 4–8 °C. At the end of the period, drug content, swelling index, surface pH, and release profiles were determined.

## RESULTS AND DISCUSSION

Ondansetron film formulations were designed with the objective of immediate release of the drug for improved patient compliance and better bioavailability. Therefore, rapidly water-soluble polymers such as PVA and PVP were chosen for the formulation with Carbopol for conferring mucoadhesive properties.

All the fabricated film formulations prepared were smooth, almost transparent with good flexibility. It was observed that result of uniformity of weight; thickness and drug content were satisfactory with respect to variation of these parameters between films of same formulation. The surface pH was close to neutral in all the formulations, and this means that they may have less potential to irritate the buccal mucosa, therefore more comfortable. All film formulations exhibited good folding endurance exceeding 300, indicating that they are tough and flexible.

**Figure 1:**
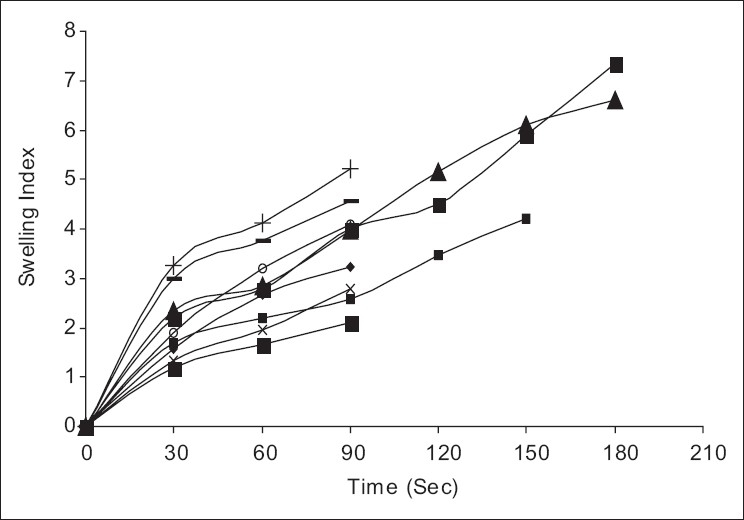
Swelling index of different formulation of fast dissolving films of ondansetron hydrochloride. (+ – +) FA_1_, (x–x) FA_2_, (–) FA_3_, (◦–◦) FB_1_, (*–*) FB_2_, (♦♦)FB_3_, (■■)FC_1_, (□□) FC_2_

The result of IR studies showed no interaction between drug, polymer, or excipients with in the formulations. The swelling index evaluation indicated that the formulations containing mannitol, i.e., FA_2,_ FB_2,_ FC_2_ exhibited less extent of swelling as compared to other formulations. The reason may be due to the presence of mannitol, which is hydrophilic, since water-soluble salt and sugar increase the erosion of the polymer. From this, it is understood that the mannitol will cause the disintegration of formulation faster compared to formulation without sweetners and formulation containing sodium saccharin, which is also water-soluble salt but used in lesser concentrations as compared to mannitol. Maximum swelling was observed for the formulation containing Carbopol as compared to those formulations containing PVP as mucoadhesive polymer. The swelling index profile for different films is shown in Figure 1.

The TS gives an indication of the strength and elasticity of the film reflected by the parameters, tensile strength (TS) and elongation at break (E/B). A weak and soft polymer is characterized by a low TS and E/B; a hard and brittle polymer shows a moderate TS and low E/B; a soft and tough polymer is characterized by a moderate TS and high E/B whereas a hard and tough polymer shows a high TS and E/B. The results of TS and percentage elongation of all the formulation containing sweetners are shown in Table 3.

**Table 3:** Results of tensile strength and percentage elongation for all films

Formulation code	Tensile strength*, kg/mm^2^	% Elongation*
FA_1_	1.141 ± 0.3	103.33 ± 3.02
FA_2_	0.846 ± 0.2	93.33 ± 2.14
FA_3_	1.023 ± 0.7	100.23 ± 3.19
FB_1_	1.444 ± 0.1	165.66 ± 2.22
FB_2_	1.151 ± 0.3	156.56 ± 2.76
FB_3_	1.334 ± 0.5	160.66 ± 1.36
FC_1_	0.817 ± 0.4	136.67 ± 4.63
FC_2_	0.788 ± 0.1	126.56 ± 3.16
FC_3_	0.802 ± 0.5	132.15 ± 1.54

* Values are represented as mean ± SD and *n* = 3

The formulation containing mannitol exhibited lower TS and % elongation compared to the others. This indicates that the presence of water-soluble compound such as mannitol and sugars in higher concentration tend to make the polymer softer and less tough and therefore poorer tensile strength and percentage elongation.

From *in vitro* drug release studies, it was found that from all three set of formulation (FA, FB, and FC), it was found that formulation containing mannitol showed high-percentage release compared to others. The formulation FA_2_ showed a maximum percentage drug release of 95.69% in 360 s followed by the formulation FB_2_ and FC_2_ of 92.59% in 330 s and 85.67% in 420 s, respectively. The order of drug release in each set of formulation can be given as:

$${\mathrm{FA}}_{2\;}>\;{\mathrm{FA}}_{3}\;>\;{\mathrm{FA}}_{1}$$

$${\mathrm{FB}}_{2\;}>\;{\mathrm{FB}}_{3}\;>\;{\mathrm{FB}}_{1}$$

$${\mathrm{FC}}_{2\;}>\;{\mathrm{FC}}_{3}\;>\;{\mathrm{FC}}_{1}$$

**Figure 2:**
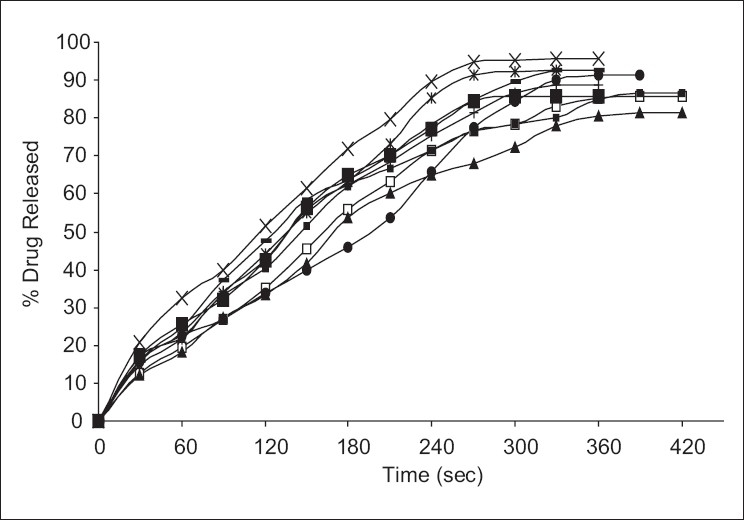
*In vitro* dissolution profile of ondansetron hydrochloride from all films. (+ - +) FA_1_, (x–x) FA_2_, (--) FA_3_, (▲▲) FB_1_, (*-*) FB_2_, (♦♦) FB_3_, (□□) FC_1_, (■■) FC_2_, (▪▪) FC_3_

The *in vitro* percentage drug release of all formulation is given in Table 4. The percentage amount of drug released is plotted against time to obtain the release profiles as shown in Figure 2.

**Table 4:** Results of *in vitro* drug release from films into simulated saliva using U.S.P Dissolution Test Apparatus I

Time (s) (Sec)	Percentage drug dissolved in simulated saliva*								
	FA			FB			FC		
	FA_1_	FA_2_	FA_3_	FB_1_	FB_2_	FB_3_	FC_1_	FC_2_	FC_3_
30	15.45 ± 1.02	20.61 ± 1.44	16.18 ± 0.10	11.99 ± 0.36	14.61 ± 0.48	15.56 ± 0.26	12.66 ± 1.19	17.23 ± 1.76	16.83 ± 0.44
60	23.31 ± 1.11	32.45 ± 0.61	24.79 ± 0.24	18.39 ± 0.29	21.65 ± 0.72	22.42 ± 1.38	19.41 ± 0.49	25.55 ± 0.08	22.26 ± 0.32
90	34.25 ± 0.75	39.63 ± 0.36	37.12 ± 0.09	27.07 ± 1.84	33.63 ± 0.27	26.67 ± 1.07	26.84 ± 0.45	32.08 ± 1.05	33.43 ± 1.11
120	42.30 ± 1.32	51.67 ± 0.24	47.42 ± 1.07	33.42 ± 0.72	44.09 ± 1.74	33.67 ± 1.28	35.27 ± 1.07	42.60 ± 2.11	40.35 ± 1.35
150	55.91 ± 0.46	61.57 ± 0.13	58.34 ± 0.77	41.71 ± 0.33	54.95 ± 1.49	40.01 ± 1.48	45.45 ± 0.75	56.15 ± 1.73	51.54 ± 0.47
180	62.22 ± 0.37	71.74 ± 0.05	63.64 ± 1.11	53.51 ± 0.22	63.23 ± 2.01	45.76 ± 1.17	55.98 ± 2.56	64.94 ± 0.82	61.85 ± 0.11
210	68.59 ± 1.62	79.83 ± 1.21	70.07 ± 0.18	60.03 ± 1.23	73.20 ± 1.23	53.76 ± 0.20	63.37 ± 1.05	70.13 ± 1.38	66.85 ± 1.09
240	75.26 ± 0.83	89.63 ± 0.17	78.50 ± 0.57	65.06 ± 0.95	85.07 ± 1.06	65.93 ± 0.09	71.62 ± 1.14	77.21 ± 1.54	71.43 ± 0.35
270	81.21 ± 0.44	94.82 ± 0.65	84.92 ± 1.08	68.01 ± 1.34	91.55 ± 0.42	77.35 ± 1.03	76.51 ± 0.87	84.44 ± 1.12	76.58 ± 0.99
300	86.73 ± 0.74	95.44 ± 0.13	89.59 ± 0.09	72.38 ± 0.45	92.40 ± 1.07	84.30 ± 0.49	78.29 ± 0.24	85.51 ± 0.32	78.29 ± 1.23
330	88.75 ± 0.78	95.66 ± 0.57	92.71 ± 1.47	77.80 ± 1.47	92.59 ± 1.11	90.23 ± 0.38	83.23 ± 1.94	85.65 ± 1.03	80.28 ± 0.24
360	88.88 ± 1.06	95.69 ± 1.13	92.82 ± 1.93	80.62 ± 1.86	–	91.33 ± 2.14	85.27 ± 0.31	85.67 ± 0.85	85.01 ± 1.08
390	–	–	–	81.19 ± 0.54	–	91.38 ± 1.95	85.71 ± 1.08	–	86.61 ± 0.25
420	–	–	–	81.27 ± 0.93	–	–	85.79 ± 0.93	–	86.71 ± 0.67

* Values are represented as mean ± SD and *n* = 3

From *ex vivo* drug release studies, it was found that formulation-containing mannitol showed highest percentage release compared to other formulations. The formulation FA_2_ showed a drug release of 79.69% in 330 s. Among the other formulations, it was found that formulation FB_2_ and FC_2_ showed better percentage drug release. The order of drug release in each set of formulation can be given as:

$${\mathrm{FA}}_{2\;}>\;{\mathrm{FA}}_{3}\;>\;{\mathrm{FA}}_{1}$$

$${\mathrm{FB}}_{2\;}>\;{\mathrm{FB}}_{3}\;>\;{\mathrm{FB}}_{1}$$

$${\mathrm{FC}}_{2\;}>\;{\mathrm{FC}}_{3}\;>\;{\mathrm{FC}}_{1}$$

**Figure 3:**
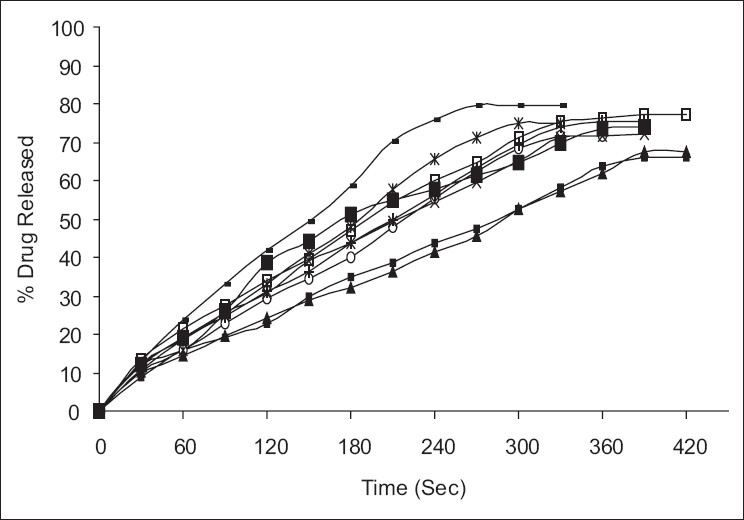
*Ex vivo* drug release from all formulations into simulated saliva. (+ - +) FA_1_, (--) FA_2_ (□□) FA_3_, (x-x) FB_1_, (*-*)FB_2_, (■■) FB_3_, (▪▪) FC_1_, (◦◦) FC_2_, (▲▲) FC_3_

The percentage amount of drug released is plotted against time to obtain the release profiles as shown in the Figure 3. During the *ex vivo* drug release study, the residence time for the formulation, i.e., the time taken for the patch to detach or erode completely from the mucosa was observed, and it was found that the FC films took longer time, probably due to their carbopol content.

**Figure 4:**
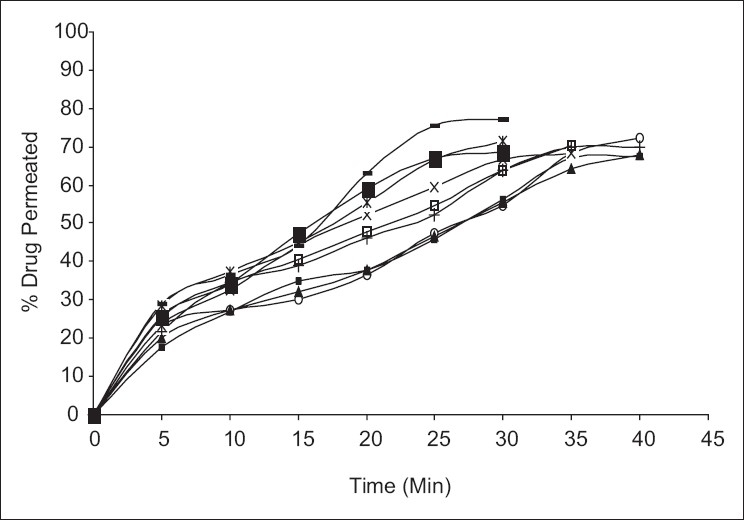
Permeation profile of ondansetron hydrochloride from all formulation through porcine buccal mucosa. (+ - +) FA_1_, (--) FA_2,_ (□□) FA_3_, (x-x) FB_1_, (*-*) FB_2_, (■■)FB_3_, (▪▪) FC_1_, (◦◦) FC_2_, (▲▲) FC_3_.

Permeation studies through oral mucosa indicated that the extent of permeation of ondansetron hydrochloride from formulations containing mannitol was observed to be much higher than other formulation. The formulation FA_2_ showed the highest percentage of drug permeated, i.e., 77.01% in 30 min. The percentage drug permeated was plotted against the time to obtain the profiles as shown in Figure 4.

The results of stability studies of ondansetron hydrochloride fast dissolving films showed no significant change with respect to drug content, surface pH, swelling index, and *in vitro* drug release at the end of 8 weeks when stored in above-mentioned conditions.

## CONCLUSION

The use of water-soluble sweeteners, especially mannitol not only enhanced the taste of the ondansetron containing films, but also increased drug release and drug permeation through oral mucosa. On the basis of data obtained from *in vitro* dissolution and *ex vivo* permeation studies that FA_2_ and FB_2_ are promising formulations suitable for the immediate release of ondansetron for systemic use since they exhibited maximum drug release and permeation, respectively.
